# Supplementary material for: Time, momentum, and energy resolved pump-probe tunneling spectroscopy of two-dimensional electron systems
Source: Nat Commun. 2023 Nov 17;14:7440. doi: 10.1038/s41467-023-43268-1 (PMC10656415; doi:10.1038/s41467-023-43268-1)
Supplement: Supplementary file 1 — Supplementary Information [file 41467_2023_43268_MOESM1_ESM.pdf]

Supplementary Note 1 - Additional discussion on temperature dependence of relaxation time.

At an elevated temperature, the 2DES at $\nu = 1$ becomes demagnetized. As a result, vacancies are created in the spin-up states in the lower Landau level. Therefore, we considered two possible decay channels for spin-up electrons in the $N = 1$ LL. First, the spin-up electrons can decay to the spin-down states in the $N = 0$ LL through a spin-flip relaxation process: $\tau^{spin\text{-}flip} = \alpha\times(1 - \rho^{spin\text{-}up})$, where $\rho^{spin\text{-}up}$ is the number of vacancies in the spin-up states in the $N = 0$ LL. Second, the spin-up electrons can decay to vacancies in the spin-up states in the $N = 0$ LL through a charge relaxation process that does not require the spin-flip: $\tau^{charge} = \beta\times\rho^{spin\text{-}up}$. The number of vacancies is proportional to $1 - M(T)$, where $M(T) = (1 - e^{-\Delta_0/(k_B T)})$ is the temperature dependent magnetization of a 2DES[1] (Δ_0, α, and β are the fitting parameters). The black dashed line in Supplementary Figure 1 shows the total relaxation time, which is the reciprocal sum of the two relaxation times $\tau^{total} = [1/(\tau^{charge})+1/(\tau^{spin\text{-}flip})]^{-1}$.

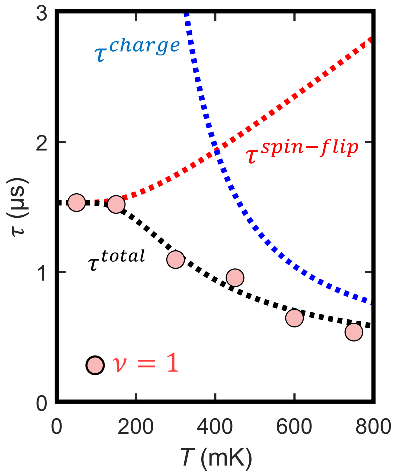

Supplementary Figure 1: Two-channel model for relaxation of pumped electrons at $\nu = 1$. Red circles show the temperature dependence of τ at $\nu = 1$ and $B_\perp = 6.0$ T. Blue, red, and black dashed lines show charge (τ^{charge}), spin-flip ($\tau^{spin\text{-}flip}$), and total decay ($\tau^{total} = [1/(\tau^{charge})+1/(\tau^{spin\text{-}flip})]^{-1}$) curves that give a best fit to the data at $\nu = 1$.

Supplementary Note 2 - Spectral function of the ν = 1+ state

We consider the spectral function characterizing the injection of an electron from the source quantum well into the second Landau level N=1 of the target quantum well while the first N=0 Landau level is occupied with electrons. In the symmetric gauge, the single-particle energies are those of a two-dimensional harmonic oscillator, with energies $E(N,m,\sigma)=\hbar\omega_c(N+\frac{1}{2})+\frac{1}{2}g\mu_B B\sigma$, where N enumerates the Landau levels (LLs), m are intra-Landau level quantum numbers and $\sigma = \pm 1$ is the electron spin. The single-particle states are characterized by the angular momentum $l = m - N$ and the cyclotron energy $\hbar\omega_c = eB/m^*$, with $\hbar$ being the reduced Planck's constant, $e > 0$ - the elementary charge, B – the magnetic field, and m^* – the electron effective mass. Further, g is the electron Landé factor (negative for GaAs), and μ_B is the Bohr magneton.

Defining $c^+_{i,\sigma}(c_{i,\sigma})$ as the operator describing the creation (annihilation) of an electron in orbital i with spin σ, the many-body Hamiltonian describing n electrons in the lowest Landau level is

$$\hat{H} = \Sigma_{i,\sigma}E(i,\sigma)c^+_{i,\sigma}c_{i,\sigma} + \frac{1}{2}\Sigma_{i,j,k}\Sigma_{\sigma,\sigma'}\langle i,j|V|k,l\rangle c^+_{i,\sigma}c^+_{j,\sigma'}c_{k,\sigma}c_{l,\sigma'} \qquad \text{(Supplementary Equation 1)}$$

Here, $\langle i,j|V|k,l\rangle$ are the Coulomb matrix elements computed in the basis of the two-dimensional harmonic oscillator states[2]. We will express all interaction energies in our system in terms of the exchange energy $E_{ex} = \sum_{i=1}^{\infty}\langle 00,0i|V|00,0i\rangle$, which gives the total exchange interaction of an electron in the orbital (0,0) with all spin-polarized electrons in the lowest LL. To make the system electrically neutral, we account for the positive background by distributing n fixed positive charges on orbitals similar to the electronic ones. We neglect the Zeeman energy in this model.

We assume that we are adding a spin-up electron to the central orbital (N, m) = (1, 1) of the second Landau level, while the remaining n electrons occupy the lowest N=0 Landau level with spin up. Our goal is to compute the spectral function of the added electron as

$$A(E) = \sum_I P_I \sum_F \left|\langle F|c^+_{1,1,\uparrow}|I\rangle\right|^2 \delta(E_F - E_I - E). \qquad \text{(Supplementary Equation 2)}$$

Here, the index I enumerates the initial states, i.e., the correlated states of interacting n electrons in the lowest LL. P_I denotes the occupation probability of the state I of the initial system, and E_I is the energy of that state. $P_I = \frac{1}{Z}e^{-E_I/k_BT}$ $Z = \sum_I e^{-E_I/k_BT}$ The index F enumerates the final states, i.e., the correlated states of interacting n+1 electrons, of which exactly one is in the second LL.

We start by computing the low-energy initial states of the n-electron system using the exact diagonalization technique in a limited basis, with variable number of electrons n. We carry out two calculations. In the first part, at early times in spin injection, we consider the addition of the spin-up electron to the second LL in the presence of the ν = 1 electronic droplet. In the second

part, at later times in injection pulse, a fraction of the electrons relax from the N=1 spin-up Landau level to the spin-down N=0 Landau level due to spin-flip scattering. We then compute the spectral function for addition of a spin-up electron to the N=1 level in the presence of spin-up electrons in the lowest LL, along with a low density of spin down electrons.

The initial states of the $\nu = 1$ system are considered by taking $n = 8$ electrons and distributing them on eight spin-up orbitals in the lowest LL. We construct the spin polarized ground state $|\nu=1\rangle = \prod_{i=1}^{n-1} c_{i,\uparrow}^{+}|0\rangle$, and spin-wave excitations from the ground state of the form $|i,j\rangle = c_{i,\downarrow}^{+} c_{j,\uparrow}|\nu=1\rangle$ [3,4]. We write our Hamiltonian as a matrix in the basis of all these configurations and diagonalize it numerically to obtain the energies and eigenstates of the initial system. As expected, we find the ground state of the $\nu = 1$ system to be the maximally polarized, ferromagnetic state.

The final states are considered in a similar way, but with an extra electron (the n+1st electron) in the second LL, that is, $c_{1,m,\uparrow}^{+}|\nu=1\rangle$. Even though the extra electron can be in principle added anywhere in the second LL, we choose to focus on m=1 as a representative subspace. The diagonalization of the Hamiltonian in this basis gives us the correlated final states. As the last step, we use the initial and final states to compute the spectral function $A(E)$ as described above. The resulting probability of injection of the spin-up electron into the second LL as a function of the energy E is shown in Fig. 4b in the main text (see the red line). For a noninteracting system, we would expect the addition peak at the cyclotron energy $E = \hbar\omega_c$, which is taken here to be equal to E_{ex}. The inclusion of interactions leads to a shift of the addition peak towards the lower energy. In simplest terms, that energy corresponds to the self-energy of the spin-up electron added to the second LL in the presence of fully occupied and spin-polarized lowest LL. We find further that the extra electron in the second LL leads to relatively little mixing between different configurations of the $\nu = 1$ system. Indeed, in our more extended calculations (not shown), in spite of taking a large number of configurations in the initial (several hundred) and final (several thousand) states, the addition signature appears consistently at roughly the same energy of order of $0.5E_{ex}$.

In the second part of the calculation, we account for an extra spin down electron which relaxed from the N=1 to N=0 (lowest) LL, that is, a spin-down electron added to the $\nu = 1$ spin-up state. For our calculations, we take $n = 9$ electrons distributed in the lowest LL on eight orbitals. We map out the lowest energy states of such a correlated system as a function of the total angular momentum and the total spin projection S_z. The results are presented in Fig. 4a of the main text. We find that the global ground state corresponds to a fully depolarized system (spin polarization 1/2, shown in black) with total angular momentum of 31. We identify this state with the correlated skyrmion |s>[5–7]. Similar spin depolarizations in droplets in the vicinity of the $\nu = 1$ state have been observed in quantum dots confining many electrons[8,9]. The state with maximal spin polarization of 7/2 is found at a higher energy (shown in blue). In the simplest terms, this state can be approximated as $|\nu = 1+> \; = c_{0,m,\downarrow}^{+}|\nu = 1>$, i.e., an uncorrelated state obtained from $\nu = 1$ by adding one spin-down electron to it.

We follow with the numerical study of the system of $n = 10$ electrons, of which exactly one occupies an orbital from the second LL. Owing to the depolarized and correlated nature of the states of $\nu = 1$ plus one electron, we have to account for all possible spin polarization and all possible angular momenta, resulting in several tens of Hilbert spaces, each of the size of hundreds to thousands of configurations (depending chiefly on spin polarization). Systematic diagonalization of all these cases gives us all necessary final states entering the calculation of $A(E)$.

We compute the spectral function assuming the skyrmion |s> as well as the maximally polarized $|\nu = 1+>|$ as the initial state of the n=9 electron system. The results are presented in Supplementary Figure 2 with the black and blue lines, respectively. We see a significant difference between these two spectral functions and that calculated for $\nu = 1$ (see Fig. 4b of the main text). Indeed, the spectral function of the skyrmion is very broad, reflecting spin depolarization processes in the $\nu = 1$ initial state, with overall blueshift from $0.5E_{ex}$ for $\nu = 1$ to $0.8E_{ex}$, for $\nu = 1+$ phase. We find therefore that the addition of a minority spin-down electron to the $\nu = 1$ spin-up electron state is reflected by the spectral function of N=1 spin-up electron blue-shifted from the spectral function of N=1 electron added to the $\nu = 1$ state. Hence, one expects a transition in the spectral function as a function of pumping density, from the peak at low energy to the peak at higher energy as spin-down electrons begin to populate the lowest spin-up Landau level.

Further details for these calculations will be presented in a future publication.

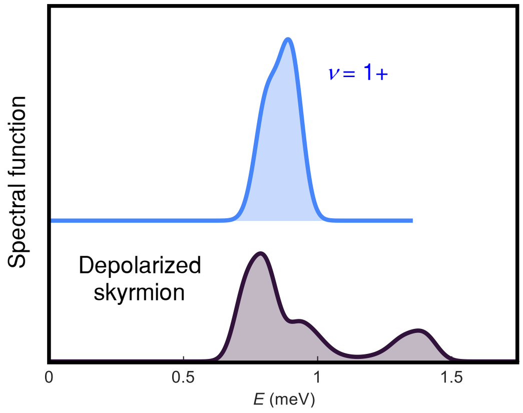

Supplementary Figure 2: Calculated spectral functions of ν = 1+ and skyrmion. The horizontal axis is the injection energy in the $N = 1$ LL in units of E_{ex}. The spectral function of a depolarized skyrmion state is broadened due to electronic correlations that give rise to nontrivial spin configurations (see Fig. 4b of the main text for the spectral functions of $\nu = 1$).

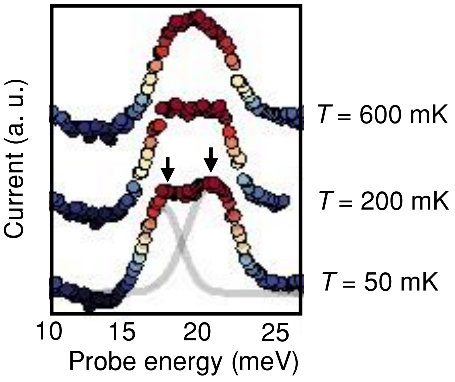

Supplementary Figure 3: Temperature dependence of the double-peak structure at $B_\perp$ = 8.0 T and Δk = 0.017 Å^{-1}. The double-peak structure gradually smoothens and disappears at elevated temperatures T ≈ 600 mK. The grey lines show the sum of two gaussian curves that best fit the data.

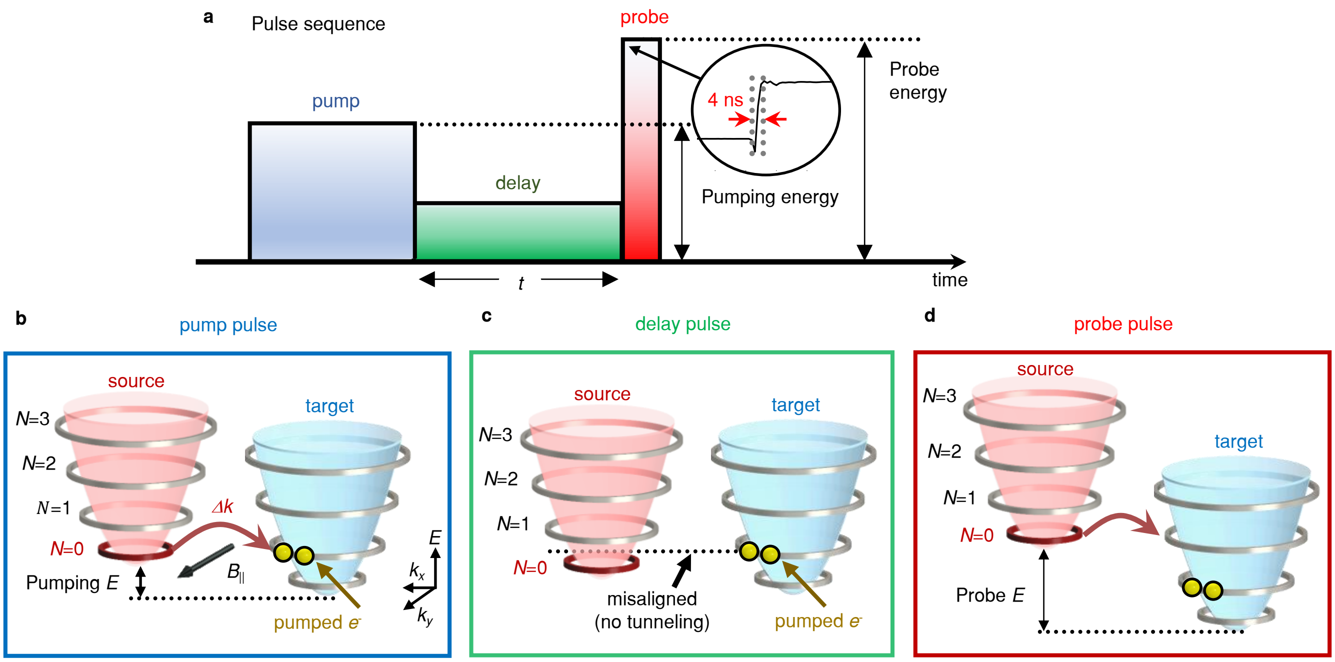

Supplementary Figure 4: Schematic representation of the pulse sequence. a, Schematic of the pump, delay, and probe pulses. The rise time of the pulses is shorter than a few nanoseconds, and all of the applied pulses do not overlap each other in time. **b-d,** Cartoons depicting the energy band diagrams of the probe and target layers when the pump, delay, and probe pulses are applied to the tunnel device. **b,** During the pump pulse, electrons in the N=0 Landau level in the source are pumped into the Landau level in the target. The height of the pump pulse selects the energy level that is being pumped. **c,** The delay pulse induces an out-of-resonance condition, in which the pumped states in the target and the unoccupied states in the source are misaligned in energy. **d,** The short probe pulse is used to measure the tunneling current flowing into the target that has been driven out of equilibrium. The energy axis in Tr-MERTS spectra in the main text is proportional to the height of the probe pulse, while the height of the pump pulse (i.e. the pumped energy level) is fixed.

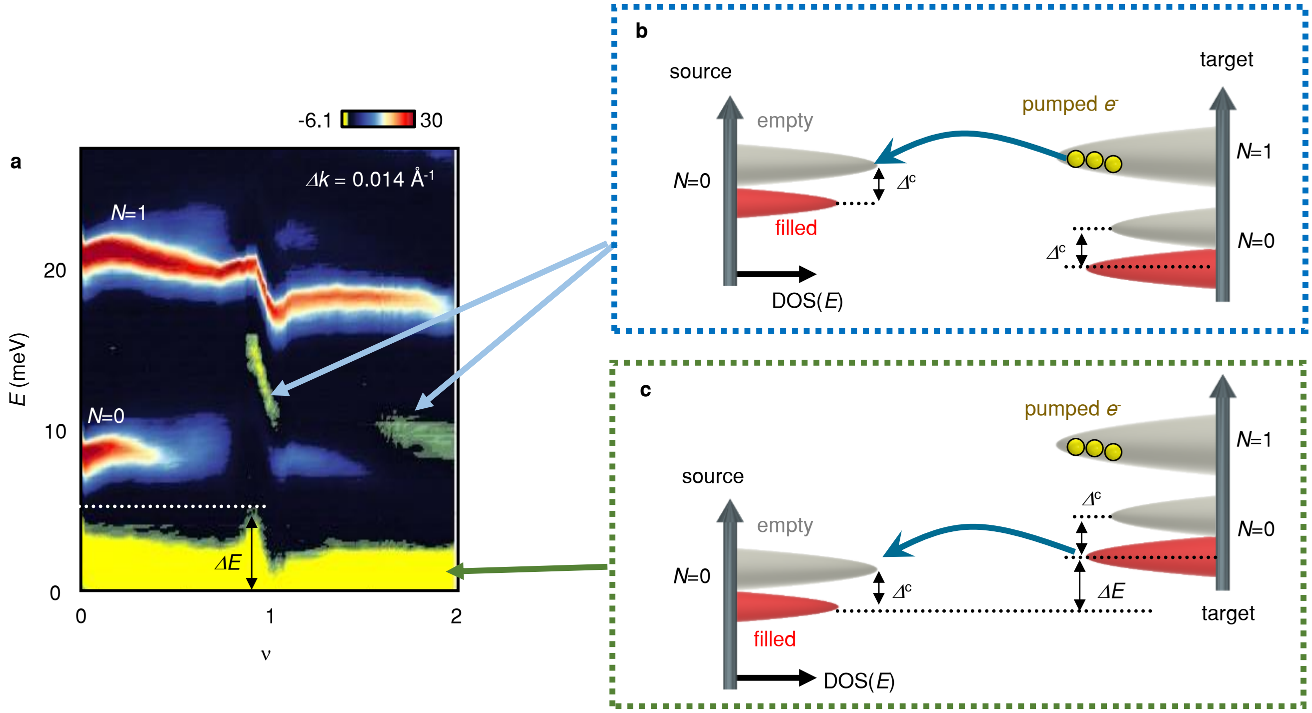

Supplementary Figure 5: A detailed explanation of the negative tunneling current observed below the N = 0 and N = 1 LLs. a, A constant Δk cut of the Tr-MERTS spectrum measured as a function of energy and filling factor. The cut is taken at t = 0, Δk = 0.014 $Å^{-1}$, at $B_{\perp}$ = 6.5 T. Yellow features below the N=1 and N=0 LLs indicate negative tunneling currents flowing back to the source quantum well under the resonance conditions depicted in (b) and (c). **b,** Negative current arises when the energy of the pumped states in the target aligns with the energy of the available states in the source, causing the pumped electrons to tunnel back to the source. Δ^c is Coulomb induced tunneling energy gap between the occupied and unoccupied states in the N = 0 Landau level. The magnitude of the transient negative current is proportional to the density of pumped electrons in the N=1 Landau level. **c,** The negative current observed below the N=0 Landau level arises due to the effect of charging the target quantum well, which causes the Landau levels in the target to shift. This shift in energy leads to the alignment of occupied states in the N=0 LL of the target and unoccupied states in the N=0 LL of the source, inducing the backflow of electrons for small probe energy pulse.

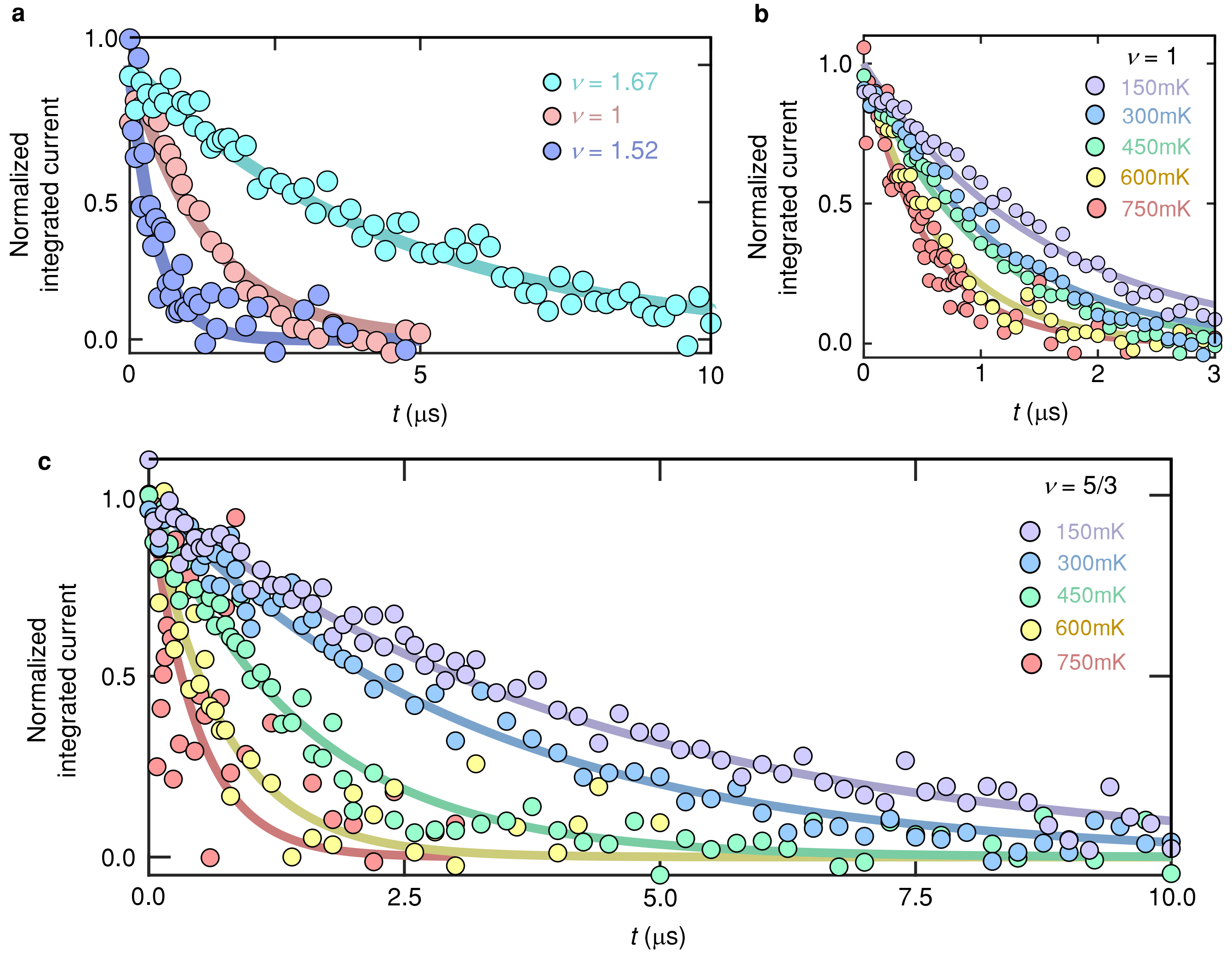

Supplementary Figure 6: Fitting exponential decay to transient current. a, Solid lines are exponential decay curves $e^{-t/\tau}$ that best fit the data (filled circles). The deduced values of τ are 1.35 μs at ν = 1, 0.45 μs at ν = 1.52, and 4.50 μs at ν = 1.67. $B_\perp$ and T are fixed at 6.5 T and 50 mK, respectively. **b**,**c**, Temperature dependence of integrated transient current at ν = 1 and 5/3. Colored solid lines are exponential decay functions that best fit the data (filled circles). The data are taken at $B_\perp$ = 6.0 T.

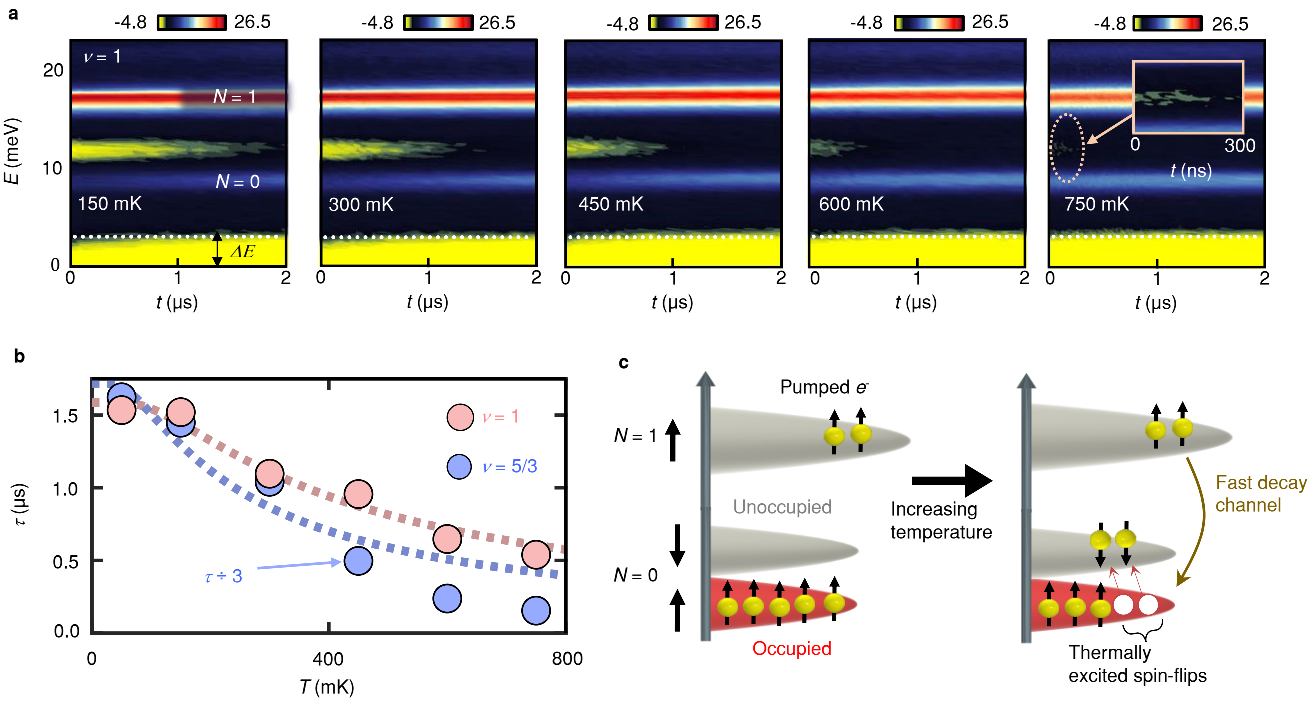

Supplementary Figure 7: *T* dependence of Tr-MERTS spectra. a, Δk = 0.013 Å^{-1} cuts at $B_\perp$ = 6.0 T and ν = 1, showing more rapid decays of injected electrons at higher T (see the decay of yellow features). **b,** T dependence of τ at ν = 1 (red) and 5/3 (blue). τ (filled circle) is determined by fitting an exponential decay to the integrated transient current (see Supplementary Figure 6). Colored dashed lines are best-fit curves assuming the demagnetization of a 2DES (see Supplementary Section 1 for details). **c,** Cartoon describing fast relaxation observed at a higher temperature. An increase in temperature creates vacancies in the majority-spin states, providing a faster decay channel for the spin-up electrons pumped into a higher LL.

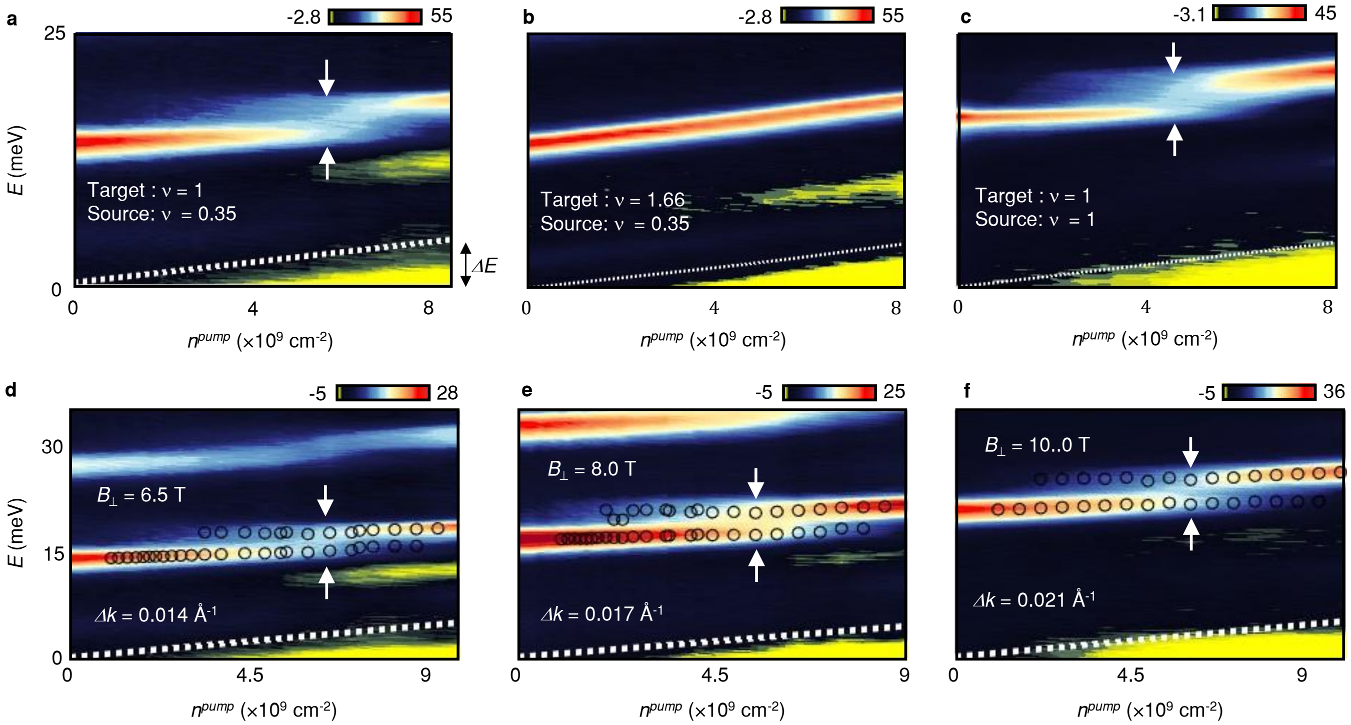

Supplementary Figure 8: Experiments demonstrating that the splitting does not depend on the filling factor of the source and occurs in a wide range of magnetic fields. a-c, Constant Δk cuts of Tr-MERTS spectrum measured as a function of n^{pump}. Each cut is measured at t = 0 and different filling factors of the target and source. The spectra are taken at $B_{\perp}$ = 6.5 T. The splitting is observed only when the filling factor of the target is ν = 1. **d-f,** Constant Δk cuts of Tr-MERTS spectrum measured at various $B_{\perp}$. Open circles indicate tunneling peaks determined by fitting a sum of two gaussian curves to the data (see Fig. 3c and 3e in the main text). The spectra are taken at T = 50 mK.

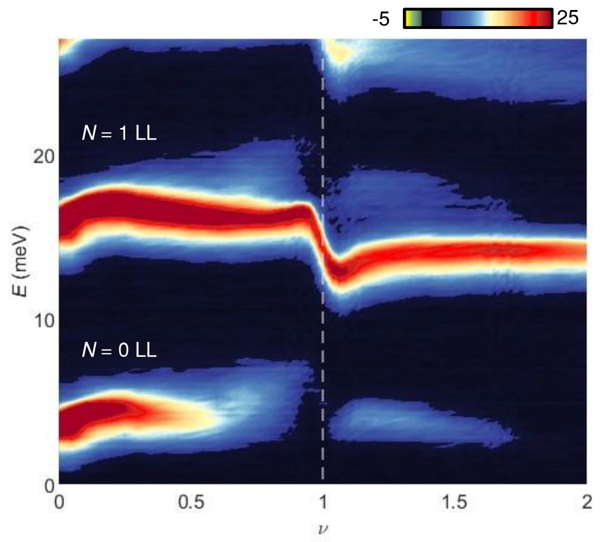

Supplementary Figure 9: Equilibrium tunneling spectrum measured without a pump pulse. The tunneling spectrum is measured at $B_{\perp}$ = 6.5 T, Δk = 0.014 Å^{-1}, and T = 50 mK. The white dashed vertical line indicates ν=1. The equilibrium spectrum does not display splitting in the N = 1 LL at ν close to 1. Note that tunneling from spin-up polarized source well into the N=0 Landau level is forbidden for ν close to 1 because there are no available empty spin-up states.

Supplementary References

1. Kasner, M. & MacDonald, A. H. Thermodynamics of Quantum Hall Ferromagnets. *Phys. Rev. Lett.* **76**, 3204–3207 (1996).
2. Hawrylak, P. & Potemski, M. Theory of photoluminescence from an interacting two-dimensional electron gas in strong magnetic fields. *Phys. Rev. B* **56**, 12386–12394 (1997).
3. Fertig, H. A., Brey, L., Côté, R. & MacDonald, A. H. Charged spin-texture excitations and the Hartree-Fock approximation in the quantum Hall effect. *Phys. Rev. B* **50**, 11018–11021 (1994).
4. Yang, K. & MacDonald, A. H. Charged pseudospin textures in double-layer quantum Hall systems with spontaneous interlayer coherence. *Phys. Rev. B* **51**, 17247–17250 (1995).
5. Yoo, H. M., Baldwin, K. W., West, K., Pfeiffer, L. & Ashoori, R. C. Spin phase diagram of the interacting quantum Hall liquid. *Nat. Phys.* 1–6 (2020).
6. Barrett, S. E., Dabbagh, G., Pfeiffer, L. N., West, K. W. & Tycko, R. Optically Pumped NMR Evidence for Finite-Size Skyrmions in GaAs Quantum Wells near Landau Level Filling ν=1. *Phys. Rev. Lett.* **74**, 5112–5115 (1995).
7. Plochocka, P. *et al.* Optical Absorption to Probe the Quantum Hall Ferromagnet at Filling Factor ν=1. *Phys. Rev. Lett.* **102**, 126806 (2009).
8. Hawrylak, P., Gould, C., Sachrajda, A., Feng, Y. & Wasilewski, Z. Collapse of the Zeeman gap in quantum dots due to electronic correlations. *Phys. Rev. B* **59**, 2801–2806 (1999).
9. Wójs, A. & Hawrylak, P. Spectral functions of quantum dots in the integer and fractional quantum Hall regime. *Phys. Rev. B* **56**, 13227–13234 (1997).
